# The Efficacy and Safety of Continuous Intravenous Endostar Treatment Combined With Concurrent Chemoradiotherapy in Patients With Locally Advanced Cervical Squamous Cell Carcinoma: A Randomized Controlled Trial

**DOI:** 10.3389/fonc.2021.723193

**Published:** 2021-08-13

**Authors:** Hang Shu, Yaqin Dong, Zhonghua Xu, Weiwei Luo, Lei Xu, Haochen Zhu, Linghui Cheng, Yin Lv

**Affiliations:** ^1^Department of Oncology Radiotherapy, The First Affiliated Hospital of Anhui Medical University, Hefei, China; ^2^Department of Obstetrics and Gynecology, First Affiliated Hospital, Anhui Medical University, Hefei, China

**Keywords:** locally advanced cervical cancer, endostar, concurrent chemoradiotherapy, short-term efficacy, safety

## Abstract

**Objective:**

To investigate the short-term efficacy and safety of Endostar combined with concurrent chemoradiotherapy in the treatment of locally advanced cervical squamous cell carcinoma (LACSC).

**Methods:**

A total of 91 patients with LACSC admitted to the First Affiliated Hospital of Anhui Medical University from June 2019 to December 2020 were randomly assigned to either the experimental group (n = 48) or control group (n = 43). The control group received radiotherapy for cervical cancer and paclitaxel combined with platinum chemotherapy (CCRT), and the experimental group received Endostar continuous intravenous infusion of anti-angiogenic therapy plus CCRT. The short-term efficacy, common clinical indicators, tumor indicators, changes in serum vascular endothelial growth factor-A (VEGF-A), and the occurrence of adverse events (AEs) were explored after treatment.

**Results:**

Compared with the control group, the complete response (CR) rate in the experimental group was significantly increased (83.33% *vs* 65.12%, P < 0.05). Both routine indicators and tumor indicators in the two groups were significantly decreased compared to before treatment. Compared with the control group, patients in the experimental group had higher incidences of neutropenia, hypertension, and infection, but lower incidence of nausea. After treatment, the serological expression of VEGF-A was significantly decreased in both groups.

**Conclusion:**

Endostar combined with CCRT in the treatment of LACSC can further improve the efficacy of CR rate and significantly reduce serum tumor indicators and VEGF-A levels, with mild and controllable AEs. Endostar combined with CCRT is expected to be a new treatment regimen for LACSC.

## Introduction

Cervical cancer is a prevalent malignancy in women, ranking as the fourth most frequently diagnosed cancer and the leading cause of cancer death in women worldwide (1). Statistics show that there were approximately 570,000 new cases and 311,000 deaths globally in 2018 (1). Although the prevention and screening techniques of cervical cancer have improved, patients are being diagnosed with cervical cancer at a younger age (2). Cervical squamous cell carcinoma remains the major pathologic type, although the HPV vaccine has led to a decrease in its incidence (3, 4). In China, there are approximately 130,000 new cases and 53,000 deaths attributed cervical cancer each year (5). Therefore, cervical cancer remains a serious threat to women’s health worldwide.

Locally advanced cervical squamous cell cancer (LACSC) refers to cervical squamous cell carcinoma with stages IB3-IVA according to the Federation International of Gynecology and Obstetrics (FIGO) classification system (2018). For patients with LACSC, concurrent chemoradiotherapy (CCRT) is the main treatment protocol, which consists of radiotherapy combined with platinum-containing chemotherapy. CCRT has become the “gold standard” treatment since the publication of five large sample, randomized controlled clinical trials conducted by the American Cancer Radiation Therapy Collegium (RTGG), the Gynecologic Oncology Group (GOG), and the Southwest Cancer Group (SWOG) (6–8). However, the 5-year overall survival (OS) rate for patients with LACSC remains only 66% (9), and within 2 years after the initial CCRT, about half of patients develop local recurrence or distant metastasis (10). Therefore, there is a need to identify new treatments for LACSC.

In 1971, Folkman proposed the hypothesis that tumor growth depends on angiogenesis (11). Many subsequent studies have confirmed that angiogenesis is the key mechanism underlying the occurrence and development of malignant tumors (12). Vascular endothelial growth factor (VEGF) and its receptor (VEGFR) have garnered much attention in the angiogenesis theory. As a monoclonal antibody to humanized VEGF, the GOG240 clinical trial showed that bevacizumab could significantly prolong the survival period of recurrent and metastatic cervical cancer, indicating that anti-angiogenic clinical treatment of cervical cancer could be beneficial. However, the incidence of adverse events (AEs) of bevacizumab, including bleeding, gastrointestinal perforation, and other adverse reactions, is very high (13, 14).

Chinese scholars developed a recombinant human vascular endostatin (Endostar; YP-16) by adding 9 amino acid sequences based on the original endostatin (15). Endostar has more stability and a longer half-life than bevacizumab and can inhibit tumor vascular growth through multiple targets. In addition, Endostar can help normalize the tumor vascular network, improve blood oxygen transport, and improve the treatment effect of radiotherapy (16). In 2005, Endostar was formally approved by the Chinese Food and Drug Administration as a first-line drug for recurrent and metastatic non-small cell lung cancer (NSCLC). In addition, Endostar is more affordable than other antiangiogenic drugs on the market, reducing the cost-burden on patients. Currently, studies involving NSCLC (17), nasopharyngeal carcinoma (NPC) (18), and bone and soft tissue sarcomas (19) have shown that clinical radiotherapy and chemotherapy have achieved a good effect when combined with Endostar. However, there are only a few reports on the efficacy and safety of Endostar combined with CCRT in the clinical treatment of LACSC.

The objective of the present study was to compare the efficacy and safety of CCRT combined with continuous intravenous pump Endostar with CCRT alone in patients with LACSC. This study provides new insights for optimal treatment of LACSC.

## Methods

The present study was a parallel, randomized, controlled clinical trial for LACSC clinical treatment. The protocol of the present study was approved by the Ethics Committee of the First Affiliated Hospital of Anhui Medical University (PJ2019-17-14). All participating patients signed informed consent before being enrolled in the study. The study protocol strictly followed the Declaration of Helsinki.

### Patients

Consecutive patients with LACSC in the Department of Oncology Radiotherapy, Anhui Medical University, were screened from July 2019 to December 2020. The inclusion criteria were as follows: 1) LACSC patients with FIGO stage IB3-IVA tumors confirmed by pathological biopsy to be inoperable cervical squamous cell carcinoma; 2) age 18 to 75 years old; 3) KPS (Karnofsky Performance Status) score ≥ 60 points or ECOG (Eastern Cooperative Oncology Group) score 0 – 2; 4) with evaluable tumor lesions; 5) no distant metastasis confirmed by imaging; 6) without serious liver, kidney, and other organ dysfunction; and 7) at least 6 months of expected survival time. The exclusion criteria were as follows: 1) patients who could not tolerate chemoradiotherapy or targeted therapy, including serious cardiovascular disease, serious liver or kidney failure, serious neurological or mental deficiency, and acute infectious diseases; 2) patients who received anti-tumor therapy previously; and 3) pregnancy and lactation patients.

### Treatment

**Figure 1:**
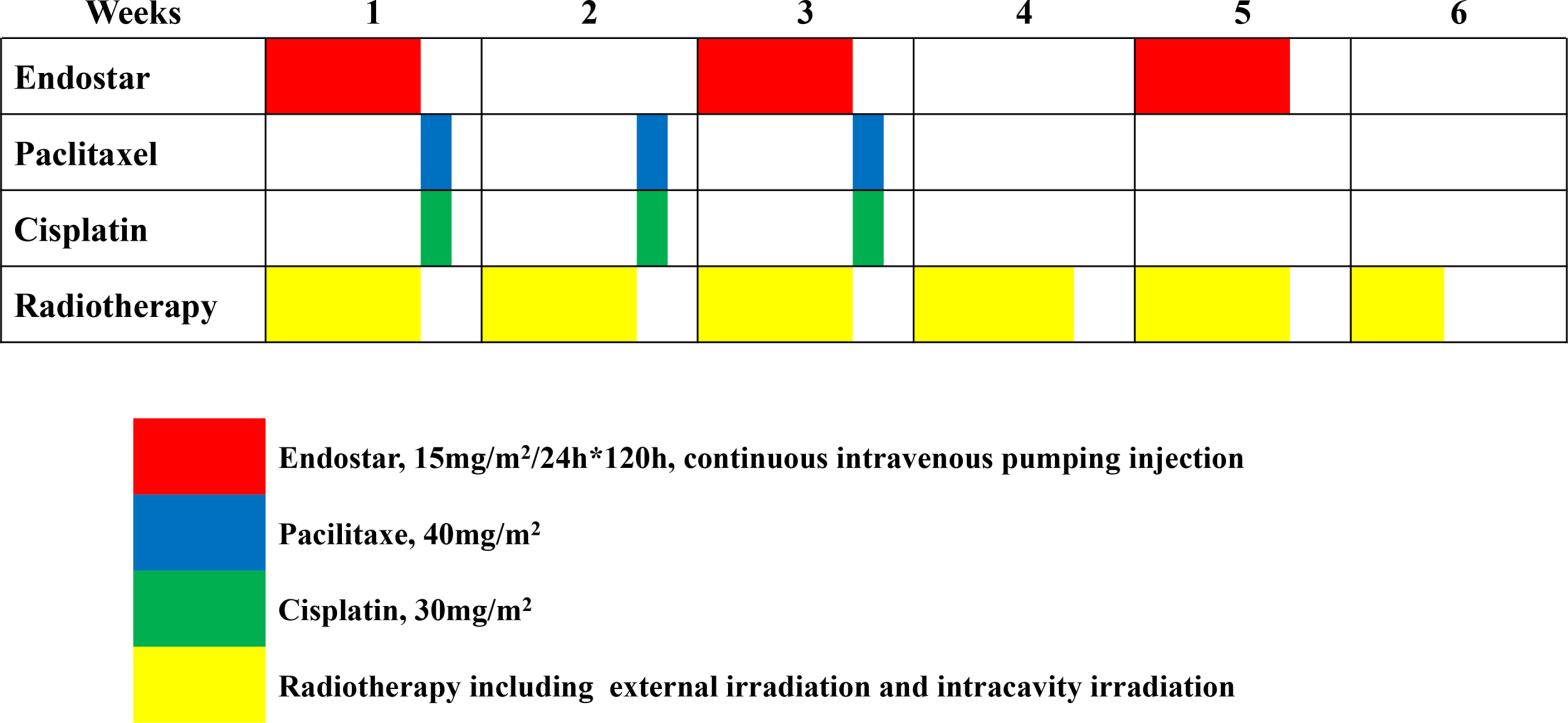
Treatment flow.

After providing informed consent, patients were randomly assigned in a 1:1 ratio to either the Endostar + CCRT arm (experimental arm) or the CCRT alone arm (control arm). The eligible patients were randomly assigned a sequence through a computer generation module to a study arm. The patient identification number was used to generate the sequence to ensure the anonymity of the assignment. Simple randomization was adopted without any restriction, such as stratification or blocking. The treatment process is shown in **Figure 1**.

### CCRT for Both the Experimental Arm and the Control Arm

All included patients received CCRT, which consisted of radiotherapy combined with platinum-containing chemotherapy. Intensity-modulated conformal radiotherapy (IMRT) was used for external irradiation. A total dose of 50 – 51 Gy/25 – 30 F was administered to the pelvic cavity and planned target area of the lymphatic drainage area (PTV, planning target volume). Patients were located using a Varian Acuity simulation locator, and images were transmitted to the ELIPSE13.6 system. All involved radiotherapists received universal training to ensure the uniform standard of target area mapping. If imaging indicated positive metastatic lymph nodes in the para-aortic or pelvic cavity, the metastatic lymph nodes were administered PTVND (planning target volume of the metastatic lymph nodes) 60 Gy/30 F. For tumors larger than 4 cm, PTVG (planning target volume of gross tumor) was added to 60 Gy/30 F simultaneously. Intracavitary retro loading radiotherapy was performed at the dose of 30 Gy/5 F. The cumulative dose of point A was ≥ 80 – 84 Gy for intracavitary retro loading therapy and external irradiation. Synchronous chemotherapy started from the first week of concurrent chemoradiotherapy with the TP regimen of paclitaxel (40 mg/m^2^, continuous intravenous infusion for more than 60 min) and Cisplatin (30 mg/m^2^, continuous intravenous infusion for 30 – 60 min). Chemotherapy was administered once a week for 3 weeks. Routine prophylactic use of antiemetic drugs was provided during chemotherapy.

### Endostar Therapy for the Experimental Arm

Anti-angiogenic therapy was performed with Endostar (Simcere Pharmaceutical, Nanjing, China) 15 mg/m^2^, continuous intravenous pumping for 120 h (day 1 to 5 of the week of administration, coinciding with the time of weekly radiotherapy), with repeated administration every other week for a total of 3 cycles.

### Endpoints and Assessment

The primary endpoint was short-term efficacy evaluated by the complete response (CR), partial response (PR), stable disease (SD), disease progression (PD), objective response rate (ORR), and disease control rate (DCR), and drug safety was evaluated by AEs. The CR, PR, SD, and PD were defined according to RECIST 1.1 (20). The equations for calculating ORR and DCR were ORR = (CR+PR)/total cases × 100% and DCR = (CR+PR+SD)/total cases × 100%, respectively. The short-term efficacy was evaluated by imaging all patients one month after assigned therapy. Therapy-related AEs, including drug-related and radiological AEs, during treatment were evaluated weekly in patients. The incidence of drug-related AEs was evaluated according to the International Cancer Organization Common Adverse Reactions Standard (NCI-CTCAE) 4.0, which were classified into Grades 1 - 5.

The secondary endpoints included blood indicators and VEGF-A level. Before treatment and one month after treatment, blood indicators were examined respectively in the two groups. Blood indicators included tumor markers and routine markers tested in all participants both before and after the interventions. Tumor markers included squamous cell carcinoma antigen (SCC), carcinoembryonic antigen (CEA), and cytokeratin 19 fragments (CYFR21-1), which were detected using chemiluminescence. Routine indicators included white blood cell (WBC), hemoglobin (Hb), platelet (PLT), and albumin (ALB). Among the members of the VEGF family, VEGF-A plays the most important role in promoting blood vessel growth (21). In this study, serum VEGF-A levels were detected by timely extraction of venous blood from patients within 24 h before and after treatment. Fasting venous blood (3 ml) was collected from the patients after and before treatment for all included patients, which was centrifuged at 2,500 r/min for 10 min. VEGF-A levels were detected in the supernatant using enzyme-linked immunosorbent assay (ELISA, Jiangsu Enzyme Industrial Co. Ltd.).

### Trial Oversight

To ensure the objectivity, authenticity, and reliability of the clinical trial, the sponsor assigned its inspectors to evaluate the study parameters regularly, supervise the case report form, check its qualification, and put forward suggestions for improvement.

### Statistical Analysis

SPSS 24.0 statistical software was used for all data analysis. Continuous variables conforming to normal distribution are expressed as mean ± standard deviation (SD) and analyzed by t-test. Median and quaternary values (P_25_, P_75_) were used for expression data that did not conform to the normal distribution, and a rank-sum test (Z test) was used for comparison. Categorical variables are described with count and frequency. The rates of the two groups were compared using the Chi-square analysis or the Fisher’s exact probability method. Logistic regression was used to explore the independent factors for efficacy and safety. P < 0.05 indicated statistical significance.

## Results

### Patients

Of the 95 patients screened, 4 patients did not meet the eligibility criteria due to missing the follow-up. Thus, 91 patients underwent randomization; 48 patients were enrolled in the experimental group and 43 patients in the control group. Each enrolled patient completed the evaluation of short-term efficacy, therapy-related AEs, blood indicators, and VEGF-A levels. By the end of the study, no patients had recurrence or died during follow-up. Each patient was followed up from the beginning of treatment to one month after the end of treatment. Therefore, the long-term survival data were not available. There were no statistical differences between the groups in terms of the baseline characteristics including age, height, weight, body surface area, BMI, level of education, menopause, basic diseases, clinical stage, and tumor size. See **Table 1** for details.

**Table 1:** Comparison of baseline characteristics between the two groups.

Clinical features	Control group (n=43)	Experimental group (n=48)	t/χ^2^	P value
**Age (years), mean ± SD**	56.49 ± 11.65	56.52 ± 10.49	0.14	0.989
**Height (cm), mean ± SD**	157.98 ± 6.10	159.11 ± 4.95	0.982	0.329
**Weight (kg), mean ± SD**	56.59 ± 9.10	58.01 ± 10.86	0.648	0.519
**Body surface area (m^2^)**	1.66 ± 0.14	1.68 ± 0.16	0.831	0.408
**BMI**	22.70 ± 3.94	22.87 ± 3.77	0.207	0.836
**Level of education**			0.729	0.393
Illiteracy	19	17		
Primary education or above	24	31		
**Menopause**			0.025	0.875
After	24	26		
Before	19	22		
**Basis of disease (hypertension, diabetes, etc.)**			0.288	0.591
No	32	38		
Yes	11	10		
**FIGO stage**			4.041	0.401
IIB	20	20		
IIIA	3	4		
IIIB	3	10		
IIIC	13	10		
IVA	4	4		
**Tumor size (cm)**			0.282	0.596
<4	20	25		
4 or higher	23	23		

SD, standard deviation; BMI, body mass index; FIGO, International Federation of Gynecology and Obstetrics.

### Short-Term Efficacy

The experimental group achieved CR 83.33% (40/48), ORR 93.75% (45/48), and DCR 95.83% (46/48), while the control group achieved CR 65.12% (28/43), ORR 39 90.70% (39/43), and DCR 95.35% (41/43). The CR rate was statistically different between the two groups (P < 0.05). No statistical difference was found for ORR and DCR (P > 0.05). The detailed comparison of the short-term efficacy is described in **Table 2**.

**Table 2:** Comparison of short-term efficacy between the two groups after treatment.

Short term efficacy	Control group (n=43)	Experimental group (n=48)	P value (chi-square test or Fisher’s exact test)
**CR, N (%)**	28 (65.12)	40 (83.33)	0.046
**PR, N (%)**	11 (22.92)	5 (11.63)	0.058
**SD, N (%)**	2 (4.17)	1 (2.33)	0.601^*^
**PD, N (%)**	2 (4.17)	2 (4.65	1.00^*^
**ORR, N (%)**	39 (90.70)	45 (93.75)	0.703^*^
**DCR, N (%)**	41 (95.35)	46 (95.93)	1.00^*^

*Fisher’s exact test.

CR, complete response; PR, partial response; SD, stable disease; ORR, objective response rate; DCR, disease control rate.

### Therapy-Related AEs

Common drug-related AEs included leukopenia, neutropenia, thrombocytopenia, hematuria, proteinuria, hypertension, bleeding, infection, nausea, vomiting, and diarrhea. The incidence of neutropenia, hypertension, and infection in the experimental group was significantly higher than in the control group, but the incidence of nausea was significantly lower in the control group (all P < 0.05). Adverse reactions mostly occurred in grades 1 - 2 in the two groups. The detailed incidence with grades of each AE is described in **Table 3**.

**Table 3:** The occurrence and comparison of acute toxic and side effects between the two groups.

	Arms	Classification of acute toxic reactions					Number of cases (%)	χ^2^	*P*
		0	1	2	3	4			
leukopenia	Control	15	8	13	4	3	28 (65.12%).	1.062	0.303
	Experimental	12	14	16	6	0	36 (75.00%).		
Neutropenia	Control	24	5	7	3	4	19 (44.19%).	5.588	0.018
	Experimental	15	18	9	5	1	33 (68.75%).		
thrombocytopenia	Control	30	9	2	1	1	13 (30.23%).	0.276	0.599
	Experimental	31	12	5	0	0	17 (35.42%).		
Blood in the urine	Control	22	14	6	0	1	21 (48.84%).	0.008	0.930
	Experimental	25	16	6	1	0	23 (47.92%).		
proteinuria	Control	32	6	4	1	0	11 (25.58%).	0.653	0.419
	Experimental	32	12	4	0	0	16 (33.33%).		
hypertension	Control	42	1	0	0	0	1 (2.33%).		0.032^*^
	Experimental	40	8	0	0	0	8 (16.67%)		
bleeding	Control	34	7	2	0	0	9 (20.93%)	0.212	0.645
	Experimental	36	11	1	0	0	12 (25.00%).		
infection	Control	35	4	2	2	0	8 (18.60%)	9.806	0.02
	Experimental	24	16	8	0	0	24 (50.00%).		
nausea	Control	17	12	12	2	0	26 (60.47%).	3.957	0.046
	Experimental	29	9	10	0	0	20 (41.67%).		
vomiting	Control	28	8	5	2	0	15 (34.88%).	1.062	0.303
	Experimental	36	7	5	0	0	12 (25.00%).		
diarrhea	Control	32	6	2	3	0	11 (25.58%).	0.026	0.871
	Experimental	35	10	3	0	0	13 (27.08%).		
Skin damage^*^	Control	34	8	0	0	1	9 (20.93%)	0.052	0.819
	Experimental	37	10	1	0	0	11 (22.92%).		
Lower alimentary canal and pelvic cavity^*^	Control	35	5	2	1	0	8 (18.60%)	3.217	0.073
	Experimental	31	8	8	0	1	17 (35.42%).		
Genitourinary tract^*^	Control	40	0	2	0	1	3 (6.98%)		0.323^*^
	Experimental	41	5	1	1	0	7 (14.58%)		
Radiation cystitis^*^	Control	42	0	0	0	1	1 (2.33%).		1.00^*^
	Experimental	46	1	0	1	0	2 (4.17%).		
Radiation proctitis^*^	Control	38	3	1	1	0	5 (11.63%)	0.173	0.677
	Experimental	41	1	5	0	1	7 (14.58%)		

*denotes radiation-related injury.

We further explored the risk factors for the occurrence of neutropenia, hypertension, infection, and nausea using multivariate analysis. The results showed that CCRT combined with Endostar was an independent risk factor for the increased incidence of both neutropenia and infection; the risk of the experimental group was 2.885 and 4.455 times higher than the control group, respectively. The combination of Endostar in treatment was also an independent risk factor for reducing the risk of nausea (P = 0.011). Even when blood pressure was routinely controlled during treatment, patients with underlying diseases had a significantly further increased risk of hypertension following Endostar treatment (P = 0.004). The details of the multivariate regression are described in **Table 4**.

**Table 4:** The adjusted odds ratios through multivariate regression for clarifying the correlation between toxicological reactions and Endostar.

Variables		Neutropenia	Hypertension	Infection	Nausea
**Age**	OR (95%CI)	0.993 (0.939-1.051)	1.069 (0.951-1.203)	0.967 (0.909-1.028)	0.962 (0.910-1.019)
	P value	0.817	0.263	0.280	0.183
**Height**	OR (95%CI)	1.038 (0.944-1.141)	1.196 (0.969-1.476)	0.950 (0.858-1.052)	1.000 (0.911-1.097)
	P value	0.438	0.095	0.325	1.000
**Weight**	OR (95%CI)	0.964 (0.916-1.014)	0.957 (0.874-1.048)	1.002 (0.950-1.057)	1.016 (0.969-1.065)
	P value	0.155	0.343	0.941	0.521
**Level of education (**Illiteracy *versus* literacy)	OR (95%CI)	2.35 (0.804-6.871)	1.198 (0.148-9.697)	1.870 (0.584-5.983)	2.317 (0.777-6.909)
	P value	0.118	0.866	0.292	0.132
**Having basis of disease** (yes *versus* no)	OR (95%CI)	2.489 (0.739-8.380)	19.801 (2.663-147.237)	0.483 (0.133-1.746)	0.704 (0.226-2.197)
	P value	0.141	0.004	0.267	0.545
**Menopause** (after *versus* before)	OR (95%CI)	1.112 (0.391-3.169)	0.846 (0.098-7.312)	0.701 (0.232-2.121)	0.364 (0.124-1.072)
	P value	0.842	0.879	0.530	0.067
**FIGO stages** (IIB-IIIB *versus* IIIC-IVA)	OR (95%CI)	0.823 (0.251-2.691)	1.759 (0.102-30.364)	0.669 (0.180-2.484)	0.401 (0.119-1.349)
	P value	0.747	0.697	0.548	0.140
**Tumor size** (< 4 cm *versus* ≥4 cm)	OR (95%CI)	0.684 (0.470-3.161)	3.544 (0.541-23.207)	1.697 (0.622-4.634)	1.237 (0.487-3.145)
	P value	0.684	0.187	0.302	0.655
**Arms** (Control *versus* Experimental)	OR (95%CI)	2.885 (1.063-7.833)	9.660 (1.062-87.835)	4.455 (1.472-13.485)	0.268 (0.097-0.744)
	P value	0.038	0.044	0.008	0.011

Radiation-related AEs included skin damage, lower gastrointestinal and pelvic reactions, genitourinary tract reactions, radiation cystitis, and radiation proctitis. Most of the radiation-related AES were grades 1 - 2 (see details in **Table 3**). No statistically significant difference was found for any radiation-related AEs between the two groups (P > 0,05). The statistical data are described in **Table 3**.

### Blood Indicators and VEGF-A Levels

Among the common clinical indicators, white blood cells and platelets decreased in the Control group one month after treatment compared with before treatment (P < 0.001). However, the phenomenon did not appear in the treatment of CCRT combined with Endostar (P = 0.309). Compared with before treatment, hemoglobin in both groups decreased after treatment, and the difference was statistically significant (all P < 0.05). In terms of cervical cancer-specific tumor indexes, SCC, CEA, and CYRA21-1 in both groups significantly decreased after treatment compared with before treatment, (all P < 0.05) (**Table 5**). We also compared the range of changes in the indicators and found that there was no difference in the decrease ranges of four indicators of hemoglobin, SCC, CEA, and Cyra21-1 in the two groups (all P > 0.05) (**Supplementary Table 1**).

**Table 5:** Comparison of blood indicators and VEGF-A between the two groups before and after treatment.

	Indicators	Arms	Before the treatment	After treatment	Z/t	P value
**Routine indexes (Unit)**	WBC (x 10^9^/L)	Control	*5.29 (3.89, 6.7)	*4.11 (3.09, 4.64)	3.592	<0.001
		Experimental	^4.230 ± 1.67	^4.70 ± 2.11	1.029	0.309
	HB (g/L)	Control	*111.00 (102.00, 122.00)	*106.00 (100.00, 116.00)	3.177	0.001
		Experimental	^113.02 ± 15.68	^107.00 ± 12.64	3.092	0.003
	PLT (x 10^9^/L)	Control	^244.00 ± 103.84	^184.62 ± 70.25	4.226	<0.001
		Experimental	^174.52 ± 63.16	^158.60 ± 51.98	1.684	0.099
	ALB (g/L)	Control	*41.20 (38.10, 42.70)	*39.90 (37.10, 42.60)	1.357	0.175
		Experimental	*42.75 (40.25, 44.85)	*41.40 (38.05, 43.60)	2.101	0.36
**Tumor indexes (Unit, reference value)**	SCC (ng/L, 0-3.00)	Control	*4.45 (2.17, 8.20)	*1.72 (0.80, 5.10)	3.900	<0.001
		Experimental	*3.29 (1.27, 6.46)	*1.20 (0.77, 2.08)	5.143	<0.001
	CEA (ng/ml, 0-5.00)	Control	*3.12 (2.10, 5.10)	*1.90 (1.3, 4.30)	2.857	0.004
		Experimental	*2.86 (1.43, 5.10)	*1.68 (1.10, 2.78)	5.208	<0.001
	CYRA21-1(ng/ml, 0-3.30)	Control	*3.02 (2.19, 4.07)	*2.07 (1.56, 2.88)	2.702	0.007
		Experimental	*3.01 (1.74, 3.93)	*2.16 (1.47, 3.15)	3.069	0.002
**VEGF-A (pg/ml)**		Control	^+^285.44 ± 53.25	^+^264.18 ± 49.24	4.183	<0.001
		Experimental	295.64 ± 73.44	273.13 ± 65.60	3.030	0.004

*Median and quaternary values (P_25_, P_75_). ^mean ± standard deviation.

^+^Before and after treatment, t test was used to analyze VEGF-A levels in the two groups, and there was no statistical difference between the two groups (P > 0.05).

VEGF-A levels before treatment were 285.44 ± 53.25 and 285.44 ± 53.25 in the control and experimental groups, respectively (P > 0.05). After treatment, VEGF-A levels significantly decreased in both groups compared with before treatment (all P < 0.01). However, there was no statistically significant difference in VEGF-A levels between the two groups after treatment (P > 0.05). See details in **Table 5**.

## Discussion

As an anti-angiogenic drug, Endostar can block angiogenesis and directly kill tumor cells. Besides, Endostar can also improve systemic chemotherapy by increasing tumor perfusion (22) and optimizing the hypoxic environment to increase radiotherapy sensitivity (16). In the present study, we found Endostar combined with CCRT had a higher CR rate compared to CCRT alone (P = 0.046). Recently, Guan et al. reported a randomized controlled trial confirming Endostar’s ability to restore vascular homeostasis and enhance chemotherapy in patients with cervical cancer (15), which is consistent with the results of the present study.

In terms of the safety, we found that Endostar plus CCRT was an independent risk factor for neutropenia and infection. Similarly, it was once reported that a higher incidence of neutropenia occurred in locally advanced NSCLC when combined with Endostar (23). The high frequency of infection could be due to neutropenia, as there is a potential connection between AEs. Furthermore, Senior et al. reported that infection seemed to block the growth of blood vessels in tumors (24). This unique observation seems to support the anti-angiogenic effect we observed in the experimental group. However, in terms of increased blood pressure, patients in the experimental group had significantly increased risks of hypertension. VEGF is critical for maintaining normal blood pressure and can induce the release of nitic oxide (NO) and prostaglandin (PGI2) from endothelial cells, promoting vasodilation (25). Endostar can down-regulate VEGF expression (26). Therefore, elevated blood pressure is more common following Endostar treatment. It is worth noting that the risk of nausea was lower in the experimental group in this study, providing a new insight for clinical treatment.

The levels of white blood cells and platelets in the control group decreased after treatment, but not in the experimental group. The data suggest that Endostar, as a targeted anti-angiogenic therapy, does not cause myelosuppression in patients after long-term combined treatment with CCRT. In the present study, patients in both groups experienced a slight decrease in hemoglobin during the one-month follow-up Most patients with LACSC have tumor bleeding, and concurrent radiotherapy can also decrease hemoglobin, as previously reported (27). SCC, CEA, and CYRA21-1 are tumor markers that are clearly related to tumor burden of cervical cancer (28, 29). These tumor indexes significantly decreased in both groups after treatment in this study. The results reconfirm the efficacy of Endostar in combination with CCRT in patients with LACSC. As for VEGF-A, we did not observe an improving effect of Endostar on the inhibition of VEGF-A with CCRT treatment. A decrease in VEGF-A levels has been significantly helpful in the prolongation of patient survival following treatment of various tumors (30, 31). However, a study of head and neck squamous cell carcinoma suggested that an increase in serum VEGF-A levels is a significant negative predictor of radiotherapy or chemoradiotherapy (P = 0.001) (32). Future research is needed to further explore the effect of Endostar on VEGF-A levels.

In the past, Endostar used intermittent intravenous infusion (IIV). In this study, we administered continuous intravenous (CIV) administration over a period of 120 hours with a portable infusion pump. In terms of treatment options, Endostar has a half-life of about 10 hours (33) and IIV causes drug concentrations to fluctuate greatly, but CIV can ensure a stable blood drug concentration in the body. In contrast, patients with LACSC received conventional fractionated radiotherapy once a day, Monday through Friday. The 120-hour CIV was administered from Monday to Friday to ensure that the Endostar treatment coincided with radiotherapy. It is important to note that, theoretically, stable drug concentrations have milder toxic side effects (34) and have been reported to support a possible survival benefit (35). LACSC patients with CCRT often present with gynecological symptoms and reduced abilities to carry out activities of daily living. The CIV is delivered in a portable manner, reducing patient time costs and improving compliance. In clinical practice, traditional IIV requires more infusion devices, while portable infusion pumps can reduce the cost of medical resources and reduce the workload of nursing staff. Therefore, CIV is a suitable way to use Endostar in combination with CCRT.

As CCRT has become the “gold standard” treatment for LACSC, there have been consistent efforts to improve CCRT to achieve better efficacy. Studies have reported that 5-year OS was higher in patients with a tri-weekly cisplatin regimen compared with a traditional weekly cisplatin regimen combined with CCRT (36). Carboplatin is a viable option for patients who cannot tolerate cisplatin in CCRT (37). A phase III clinical trial in Mexico showed that following 2 cycles of adjuvant chemotherapy after CCRT, the 3-year PFS increased from 65% to 74.4% for LACSC (38). When S-1 was added to the traditional CCRT regimen, the OS and PFS of patients with LACSC improved and there was no increase in the toxic side effects (39). When CCRT is combined with autologous cytokine-induced killer cell infusion, LACSC patients have better short-term efficacy and better quality of life (40). However, studies on CCRT combined with antiangiogenic therapy in patients with LACSC are rare, and this study is expected to provide new insight.

To the best of our best, the present study is the first trial exploring the efficacy and safety of Endostar in patients with LACSC. Limitations of this study include the small sample size and lack of long-term survival data, which may lower the power of the analysis. Currently, there is clinical evidence supporting the significant survival benefit of Endostar in the treatment of NSCLC. In addition, Endostar has been included in China’s national medical insurance for the treatment of patients with advanced NSCLC. However, for other cancers, there is still a lack of broad, large clinical trials to support the benefits of Endostar, which is why Endostar is limited to the Chinese market. Therefore, more evidence is needed to determine the optimal dose, administration route, administration time window, and drug safety of Endostar. With more basic research we can better understand the potential value of Endostar in clinical application.

## Conclusion

Endostar combined with CCRT in the treatment of LACSC can further improve the efficacy of CR rate and significantly reduce serum tumor indicators and VEGF-A levels, with mild and controllable AEs. For the treatment of LACSC, Endostar combined with CCRT could be extended to a broader clinical trial and is expected to be a new treatment regimen for LACSC.

## Data Availability Statement

The raw data supporting the conclusions of this article will be made available by the authors, without undue reservation.

## Ethics Statement 

The studies involving human participants were reviewed and approved by Ethics Committee of the First Affiliated Hospital of Anhui Medical University. The patients/participants provided their written informed consent to participate in this study.

## Author Contributions

YL and LC conceived and designed the study. HS analyzed the experimental data and drafted the manuscript. ZX, LX, and HZ collected the experimental data. WL and YD revised the manuscript for important intellectual content. All authors contributed to the article and approved the submitted version.

## Funding

This work was supported by the China International Medical Foundation (CIMFz-2014-06-19413), China International Medical Foundation (CIMFz-2014-06-2102) and the Major Program of the Anhui Natural Science Foundation (KJ2016A754).

## Conflict of Interest

The authors declare that the research was conducted in the absence of any commercial or financial relationships that could be construed as a potential conflict of interest.

## Publisher’s Note

All claims expressed in this article are solely those of the authors and do not necessarily represent those of their affiliated organizations, or those of the publisher, the editors and the reviewers. Any product that may be evaluated in this article, or claim that may be made by its manufacturer, is not guaranteed or endorsed by the publisher.

## Abbreviations

AE, adverse event; ALB, Albumin; BMI, body mass index; CCRT, concurrent chemoradiotherapy; CEA, carcinoembryonic antigen; CR, complete response; CYFR21-1, cytokeratin 19 fragments; DCR, disease control rate; ELISA, enzyme-linked immunosorbent assay; FIGO, Federation International of Gynecology and Obstetrics; GOG, Gynecologic Oncology Group; Hb, Hemoglobin; IMRT, Intensity-modulated conformal radiotherapy; LACSC, Locally advanced cervical squamous cell carcinoma; NO, nitic oxide; NPC, nasopharyngeal carcinoma; ORR, objective response rate; OS, overall survival; PD, disease progression; PGI2, Prostaglandin; PLT, Platelet; PR, partial response; RTOG, Radiation therapy oncology group; SCC, squamous cell carcinoma antigen; SD, stable disease; SWOG, Southwest Cancer Group; VEGF, vascular endothelial growth factor; VEGF-A, vascular endothelial growth factor-A; VEGFR, vascular endothelial growth factor receptor; WBC, white blood cell.
