# Supplementary material for: The Efficacy and Safety of Continuous Intravenous Endostar Treatment Combined With Concurrent Chemoradiotherapy in Patients With Locally Advanced Cervical Squamous Cell Carcinoma: A Randomized Controlled Trial
Source: Front Oncol. 2021 Aug 13;11:723193. doi: 10.3389/fonc.2021.723193 (PMC8414882; doi:10.3389/fonc.2021.723193)
Supplement: Supplementary file 1 [file Table_1.doc]

**Supplementary Table 1.** Comparison of observation indexes before and after treatment between the two groups.

| **project** | **CONTROL** | **EXPERIMENTAL** | **Z** | *P* |
| --- | --- | --- | --- | --- |
| HB change | 7.00 (15.00, 1.00) | 7.00 (15.25, 2.75) | 0.441 | 0.659 |
| SCC change | 1.25 (4.85, 0.01) | 1.80 (4.60, 0.35) | 0.708 | 0.479 |
| The CEA change | 0.50 (1.89, 0.10) | 0.63 (1.83, 0.25) | 1.268 | 0.205 |
| CYRA21-1 change | 0.27 (1.03, 0.12) | 0.72 (1.82, 0.73) | 0.906 | 0.365 |
